# Flexible and Wearable Strain–Temperature Sensors Based on Chitosan/Ink Sponges

**DOI:** 10.3390/molecules28104083

**Published:** 2023-05-14

**Authors:** Xiaoying Lin, Feng Wu, Yunqing He, Mingxian Liu

**Affiliations:** Department of Materials Science and Engineering, College of Chemistry and Materials Science, Jinan University, Guangzhou 511443, China; linxiaoying0508@163.com (X.L.);

**Keywords:** chitosan, ink, photothermal, strain sensor, temperature sensor

## Abstract

A simple and economic strategy to construct a chitosan-ink carbon nanoparticle sponge sensor was proposed by freeze-drying of chitosan and Chinese ink mixture solution. The microstructure and physical properties of the composite sponges with different ratios are characterized. The interfacial compatibility of chitosan and carbon nanoparticles in ink is satisfied, and the mechanical property and porosity of chitosan was increased by the incorporation of carbon nanoparticles. Due to excellent conductivity and good photothermal conversion effect of the carbon nanoparticles in ink, the constructed flexible sponge sensor has satisfactory strain and temperature sensing performance and high sensitivity (133.05 ms). In addition, these sensors can be successfully applied to monitor the large joint movement of the human body and the movement of muscle groups near the esophagus. Dual functionally integrated sponge sensors show great potential for strain and temperature detection in real time. The prepared chitosan-ink carbon nanoparticle composite shows promising applications in wearable smart sensors.

## 1. Introduction

With the continuous development of modern science and technology, the development and application of electronic equipment has increased rapidly. Various sensors have been used in human motion detection, human–computer interaction, artificial intelligence medical care, human health detection, electronic skin, and other fields [1,2,3,4]. Sensors can sensitively monitor changes in pressure [5], temperature [6], humidity [7,8], gas [9,10], etc. Due to their excellent flexibility and reliability, flexible sensors have attracted extensive and growing interest. Flexible sensors can be directly attached to human skin or curved surfaces, and the obtained signals can accurately detect human physiological information. They are generally consistent with flexible substrates and conductive active substances. Many kinds of construction materials, including fiber, sponge, hydrogel, aerogel, rubber, film, and so on, are used for the design of the sensor [11,12,13,14,15,16,17]. Hydrogel and rubber materials have good stretchability and deformation recoverability, but the compactness of the materials leads to poor air permeability. In addition, adhering these materials directly to the skin easily causes inflammation, which greatly affects their development and application in the wearable field [18]. Conductive materials used for the sensor include carbon materials (carbon black, carbon nanotubes, and graphene) [19,20,21], metal nanowires [22], MXene [23], polypyrrole [24], etc. Most of the conductive materials are expensive, increasing the cost of flexible sensors. Therefore, cheap sensor materials are widely sought-after.

Chinese ink, as a traditional material that has been widely used in calligraphy and painting in Asian countries for thousands of years, is a mixture of carbon nanoparticles and adhesive [25,26]. Carbon nanoparticles in the ink are the products of incomplete combustion of pinewood or burning oil at a wick [27]. The ink has good water dispersion stability and fluidity, and it has strong adhesion to the surface of the materials. Chinese ink has a simple preparation process with a low price, which has been used in many fields. In modern science and technology, Chinese ink has been used in solar steam systems [28], photothermal therapy (PTT) of tumors [29], strain sensors [30,31], etc. For example, Wang et al. developed a PTT technique for tumor metastatic lymph nodes using Chinese ink [32]. Zhou et al. prepared a flexible 3D electrode with a high-density current (16.3 ± 0.5 mA cm^3^) by coating a natural loofah sponge with Chinese ink [33]. Zhang et al. used ink to prepare polydimethylhydrosiloxane-nickel fabric with excellent anti-icing and good photothermal properties, which was used in the anti-icing/icephobic project of outdoor clothing [34]. Our previous work also reported the advanced application of Chinese ink. Melamine foam was coated with Chinese ink by the dip-coating method, which exhibited excellent conductivity, Joule heat characteristics, and desalination ability [30]. In our other work, we used ink and water-based glue to prepare a flexible rubber sensor with multiple response functions, which can quickly detect temperature, humidity, and strain signals [31].

Chitosan is a kind of natural biopolymer, which is widely available and has the advantages of biocompatibility, environmental friendliness, regeneration, biodegradability, and antibacterial properties [35,36,37]. Chitosan sponge is a three-dimensional porous material with the advantages of being lightweight, having high porosity, and having a high strain range. However, the chitosan sponge has low compression resistance and a very unstable structure. Therefore, the combination of sponge and conductive material can improve mechanical properties. The conductive materials in the three-dimensional structure of the sponge contact each other under pressure, and the increased compactness drives the change of conductivity, which can be used as material for biological intelligence detection. Zheng et al. successfully prepared ultra-light aerogels containing chitosan, polyimide, and FeCl_3_ by freeze-drying and carbonization, which showed a wide range of excellent sensing sensitivity (10.28 kPa^–1^) and remarkable long-term compressive stability (1000 cycles of compression under 50% strain without deformation) [38]. Similarly, Wu et al. developed a carbon nanotube/chitosan aerogel sensor with hydrophobicity, high sensitivity, fast response, and repeatability. The sensor was prepared by freeze-drying and dip-coating methods, being immersed in graphene oxide and 1H, 1H, 2H, 2H-perfluoroctyliethoxysilane [39].

In this study, we proposed a strategy for preparing a flexible chitosan-ink carbon nanoparticle (CS-ink) composite sponge using the solution blending and freeze-drying method. The positively charged chitosan is closely connected with the negatively charged ink through electrostatic interaction, which enhances the mechanical properties of chitosan. Due to the excellent photothermal conversion effect of carbon nanoparticles, the temperature of the composite sponge can be rapidly increased to 254.7 °C within 5 s under the irradiation of a near-infrared laser (0.9 W/cm^2^). Moreover, CS-ink exhibits a negative temperature coefficient behavior and an outstanding temperature sensitivity, which can be used as a temperature sensor. Since the carbon nanoparticles in ink are conductive materials, the prepared CS-ink composite sponge can be used as a strain sensor. The sensor can sensitively detect human movement with a response time of 133.4 ms. This simple and low-cost flexible strain–temperature sensor can be applied to human motion and temperature detection, which shows promising potential in wearable devices.

## 2. Results and Discussion

### 2.1. Preparation of CS-Ink Sponge

**Figure 1:**
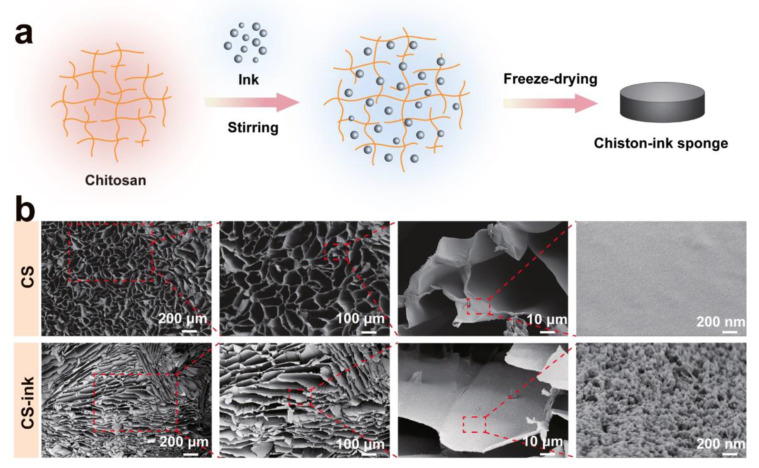
Schematic illustration of CS-ink sponge (**a**); SEM images of CS and CS-ink sponge (**b**).

Figure 1a shows the schematic of the preparation process of the chitosan-ink carbon nanoparticle sponge. Composite sponges were obtained by blending chitosan solution with ink solution and subsequently employing the freeze-drying method. Carbon nanoparticles in ink can enter the chitosan molecular network. The sponge displayed a porous three-dimensional structure, as shown in Figure 1b. With the addition of ink, the pore size of the sponge decreased. According to the SEM, the pore size of the CS-ink sponge was 34.1 ± 11.1 μm, while the size of CS was 79 ± 28.2 μm. The magnified SEM image displayed that the surface of pure chitosan was smooth, while the surface of the CS-ink sponge was covered with carbon nanoparticles uniformly. The large number of carbon nanoparticles on the surface of the chitosan suggested that the carbon nanoparticles adhered well to the surface of the chitosan, and chitosan and carbon nanoparticles have good interfacial compatibility.

### 2.2. Characterization of CS-Ink Sponge

**Figure 2:**
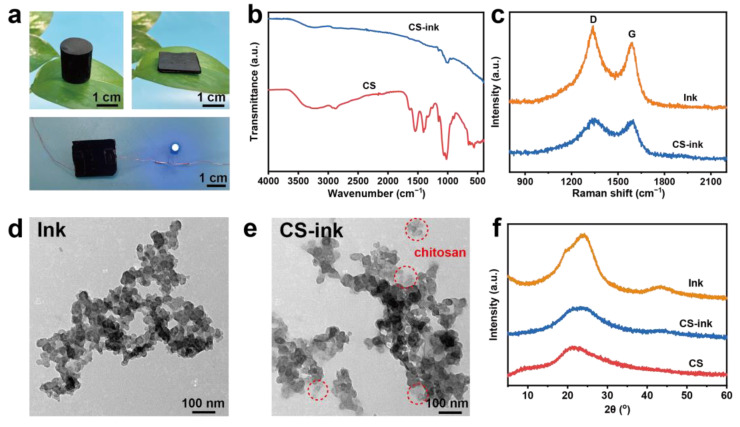
The appearance of the CS-ink sponge and the illumination of the LED bulb when linked with the CS-ink sponge in the circuit (**a**), FIRT spectrum of CS and CS-ink (**b**), Raman spectrogram of ink and CS-ink (**c**), TEM image of ink (**d**) and CS-ink (**e**), XRD patterns of CS, ink, and CS-ink (**f**).

CS-ink sponges with different shapes were shown in Figure 2a. CS-ink had a low density and can be placed on the plant leaves without bending them. The CS sponge was insulating, while the CS-ink was conductive. A circuit with a blue light-emitting diode (LED) light was designed to verify the conductivity of CS-ink. The LED lamp emitted light when the CS-ink sponge was connected to the circuit. The FTIR spectral measurement was used to explore further the composition of the CS-ink sponge (Figure 2b). The stretching vibration of the hydroxyl group (3258 cm^−1^), bending vibration of the protonated amino group (1542 cm^−1^), stretching vibration of the C–N bond (1405 cm^−1^), and the stretching vibration of the C–O bond (1018 cm^−1^) were attributed to the functional groups of pure chitosan [40]. These characteristic peaks decreased in intensity or even disappeared with the addition of ink, while the ink showed no strong absorption peaks, which were attributed to the fact that carbon nanoparticles can shield the structure information since it has a strong absorption towards the light. These results revealed that the carbon nanoparticles in ink were encapsulated in chitosan.

To further verify the combination of carbon nanoparticles and chitosan, Raman spectrum detection was performed in Figure 2c. Carbon nanoparticles in ink had a strong D band (1339 cm^−1^) and G band (1584 cm^−1^), which were typical Raman peaks of graphite-like carbon [41]. G band refers to E_2_g phonons of carbon atoms, while D band derives from in-plane defects associated with graphite carbon. These two peaks also appeared in the CS-ink sponge, proving that the characteristic bonds of the carbon nanoparticles in the composite material were not destroyed despite the addition of chitosan solution to the ink. The XRD patterns of the CS, CS-ink, and ink were illustrated in Figure 2f. There are diffraction peaks of CS-ink at 24° and 43.5°. Carbon nanoparticles broadened the peak of CS at 21.1° [42], which was influenced by (002) crystal plane of carbon. Simultaneously, CS-ink had (100) crystal plane at 43.5° [30], which was unique to the carbon nanoparticles. There was no secondary phase with any new structure in the CS-ink composite sponge. These results also suggested the successful incorporation of carbon nanoparticles with chitosan.

**Figure 3:**
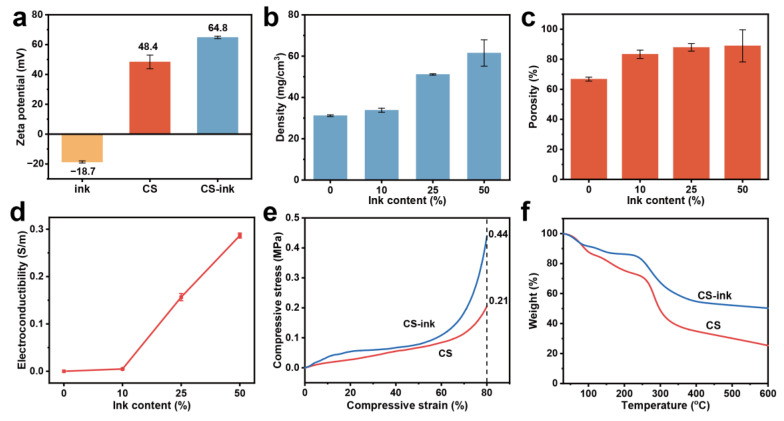
Zeta potential of ink, CS, and CS-ink (**a**); density (**b**) and porosity (**c**) of the CS-ink sponge; conductivity of the CS-ink sponge (**d**); the compressive stress–strain curves (**e**); and TG curves of CS and CS-ink sponges (**f**).

The binding of carbon nanoparticles and chitosan can be visualized in the TEM image. The morphology of carbon nanoparticles in Figure 2d was spherical, and the carbon nanoparticles in CS-ink (Figure 2e) were connected by the chitosan layer. This indicated that chitosan and carbon nanoparticles were successfully combined. Moreover, there existed electrostatic interactions between chitosan and ink. The zeta potential of ink and chitosan was −18.7 mV and +48.4 mV, respectively (Figure 3a). As expected, the zeta potential increased to +64.8 mV after the combination of ink and chitosan. The increase in zeta potential meant that the carbon nanoparticles could firmly adhere to the chitosan.

As shown in Figure 3b,c, the ink content greatly influenced the apparent density and porosity. The density of the pure chitosan sponge was 31.1 mg/cm^3^, and with the increase in ink content, the density of the sponge increased to 33.8–61.5 mg/cm^3^. The porosity of the sponge rose from 66.8% to 83.4–88.9%. It can also be seen from Figure 3d that the higher the content of ink, the greater the conductivity. The electrical conductivity of the sponges increased to 0.0047–0.2869 S/m, with the ink content increasing to 10%–50%, meaning that the addition of carbon nanoparticles can significantly improve the conductivity of the sponge.

The mechanical robustness of the sponges was investigated by the compressive stress–strain test. Figure 3e shows the representative stress–strain curve. The compressive strength of CS-ink at 80% strain was 0.44 MPa, which was 2.1 times higher than that of CS (0.21 MPa). The compressive strength of the sponge with 50% ink content reached 1.02 MPa. However, the sponge with high compressive strength was less flexible and more brittle, while those with low compressive strength tended to deform during use. Both of them were unfavorable for being used as wearable devices in sensors. Considering the flexibility and electrical conductivity of the sponge, a 1:0.25 weight ratio of chitosan solution and ink was selected as the sensor sponge in this work.

The thermal stability of the pure chitosan and the CS-ink sponge was determined by TGA, and the representative curve is shown in Figure 3f. The residual amount of CS-ink sponge was higher than that of pure chitosan in the range of 30 to 600 °C. The weight loss of CS was 74.7%, while that of the CS-ink sponge was 49.8%. Less weight loss of the sponge was attributed to the addition of ink, which also demonstrated that carbon nanoparticles facilitated the thermal stability of the sponge. Therefore, the decomposition of CS might be delayed by the incorporation of carbon nanoparticles. The loading of carbon nanoparticles was calculated to be as high as 24.9%, which was close to the ink content of 25%, indicating an effective binding of carbon nanoparticles to chitosan.

### 2.3. Photothermal Property of CS-Ink Sponge

The incorporation of carbon nanoparticles into the sponges not only endows the sponges with electrical conductivity but also with excellent photothermal conversion performance. The carbon nanoparticles in ink can effectively absorb light and convert it into heat, so the temperature of the sponge will rise with exposure to irradiation, which is known as photothermal behavior.

**Figure 4:**
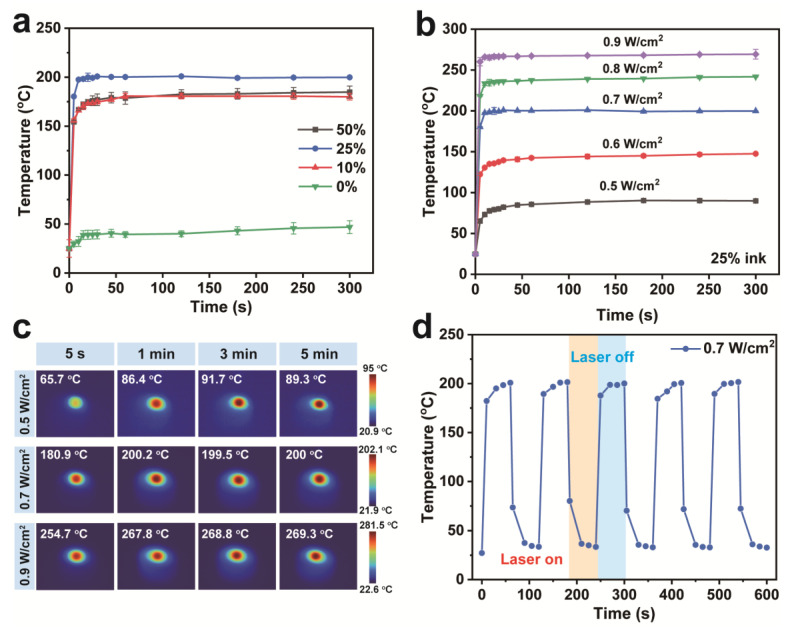
Temperature curve of the chitosan sponge with different ink content under 808 nm laser irradiation (0.7 W/cm^2^) for 5 min (**a**); temperature change (**b**) and IR thermal images (**c**) of CS-ink sponge under 808 nm laser irradiation with different power densities for 5 min; and temperature change of CS-ink sponge with an 808 nm laser switch-on/off five times (**d**).

Figure 4a showed the surface temperature variation of the sponges with different ink contents under 808 nm laser irradiation (0.7 W/cm^2^) for 5 min. At room temperature around 25 °C, the surface temperature of CS only increased by 12.6 °C. The photothermal conversion performance of the sponge was effectively improved by 10% ink. In particular, the sponge with 25% ink content had the highest photothermal effect, which was chosen as the temperature sensor. Figure 4b shows the temperature changes of the CS sponge with 25% ink contents under laser irradiation in detail. Obviously, the temperature rise was positively correlated with the laser power density, and the temperature could reach a basic equilibrium value within 60 s. Most importantly, the surface temperature could maintain a slight fluctuation at the equilibrium value with a further extension of the illumination time. Figure 4c reflected the infrared thermography of the sponge under laser irradiation of different power densities. The temperature rise was limited when the power density was 0.5 W/cm^2^. The surface temperature of the sponge can be increased to 89.3 °C within 5 min. Excitingly, the surface temperatures of sponges can sharply rise to 180.9 °C and 254.7 °C within 5 s at the power of 0.7 and 0.9 W/cm^2^, respectively. Moreover, the maximum temperature of the CS-ink was 200.2 °C and 267.8 °C under irradiation for 1 min, respectively. The photothermal stability of the CS-ink sponge was tested in Figure 4d. The photothermal effect of the CS-ink sponge can be reproduced several times when the 808 nm laser was turned on or off. Moreover, the shape of the chitosan sponge was not collapsed or deformed at the end of the cycling test. Therefore, it has been proved that CS-ink has the ultra-fast near-infrared response, efficient photothermal conversion performance, and excellent photothermal stability.

### 2.4. Application of CS-Ink Sponge as Strain Sensor

**Figure 5:**
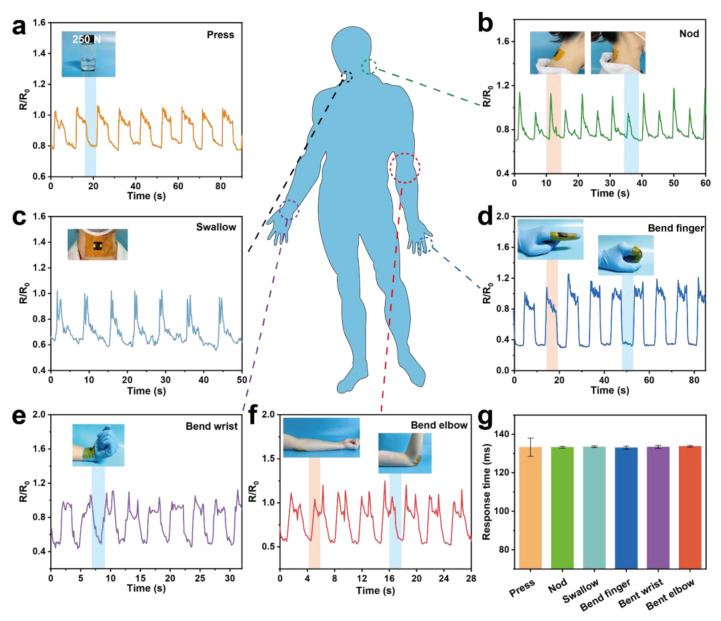
Representative resistant signal of the CS-ink sensor during pressing (**a**), nodding (**b**), swallowing (**c**), bending finger (**d**), wrist (**e**), and elbow (**f**), and response time of different movements (**g**).

Based on the superior conductivity and high porosity, CS-ink can be employed as a strain sensor. First, a weight of 250 N was pressed against the sensor (Figure 5a), and the output signal was rapid and repeatable with a response time of 133.3 ± 4.7 ms, demonstrating that the sensor can provide stable signal monitoring in real time. To evaluate the application of the sponge as a flexible strain sensor for human motion detection, the sponges were attached to the human body to sense the signal change. The sensor was attached to the cervical vertebra. As volunteers nodded their heads, the cervical vertebra drove the sponge to be stretched (Figure 5b). As a result, the relative resistance decreased. The sponge was slightly squeezed when the cervical vertebra returned to its original position. Consequently, a large peak followed by a small peak in the signal appeared throughout the monitoring process. CS-ink sensors could detect not only large movements of the human joints but also small movements of the body. As shown in Figure 5c, when the tester swallowed water, the R/R_0_ decreased sharply because the sensor was subjected to pressure from the muscle movement near the esophagus. This characteristic can be applicable to the initial diagnosis and real-time therapeutic monitoring of patients suffering from swallowing difficulties. The strain sensor was applied to the finger joint to detect the bending of the finger (Figure 5d). When the finger was bent, the conductive network in the sensor was squeezed, causing the R/R_0_ to decrease rapidly while remaining balanced. When the finger was straightened, the R/R_0_ returned to its original position. Similarly, the R/R_0_ of the sponge attached to the elbow decreased when the elbow was bent, which was also caused by the sponge being squeezed. There will be regular and periodic signal responses when bending and straightening the wrist (Figure 5e) and elbow (Figure 5f) at the same angle and frequency. As shown in Figure 5g, the response time was rapid and stable. In summary, CS-ink sensors as wearable flexible devices have the ability to monitor human movement in real time, which shows great potential for applications in medical health monitoring and motion detection.

### 2.5. Application of CS-Ink as Temperature Detection Sensor

**Figure 6:**
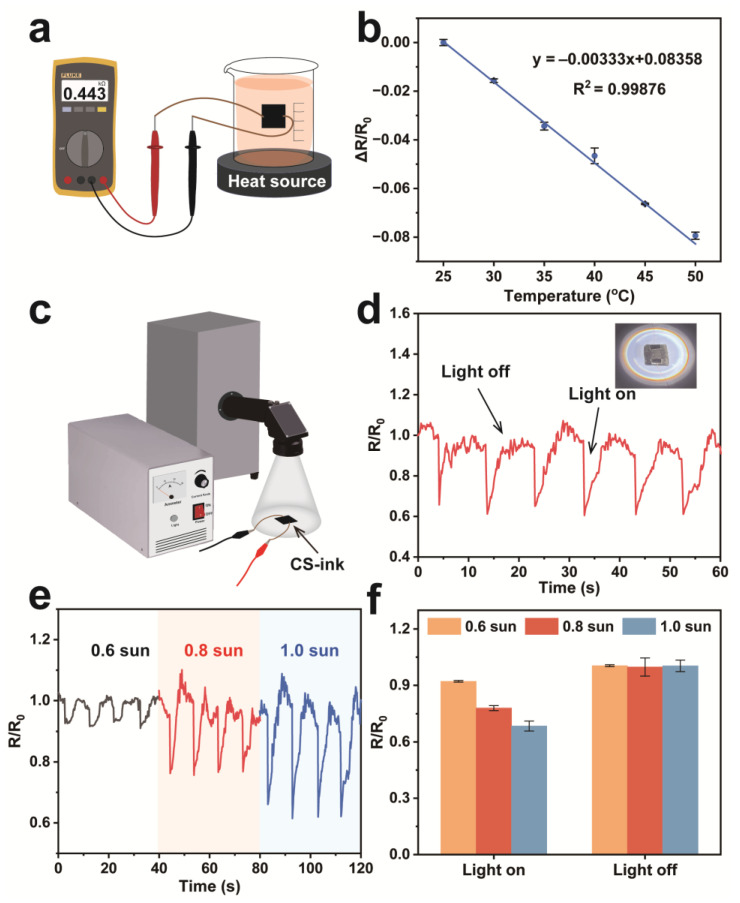
Schematic diagram of the CS-ink sensor used for temperature detection (**a**), resistance changes of the sensor during the temperature rise (**b**), schematic diagram of the test device of temperature response sensor (**c**), detection of temperature changes caused by light irradiation (**d**), resistance changes of the sensor under the irradiation of different xenon lamps (**e**), and resistance changes of the sensor when the xenon lamp was lighted on or off (**f**).

The CS-ink sponge with excellent photothermal conversion performance makes the sponge as a sensor that can be used for temperature monitoring. Firstly, the relationship of sponge resistance changing with temperature was tested. Figure 6a was a schematic diagram for detecting the temperature resistance change of the sponge from 25 to 50 °C. As can be seen from Figure 6b, ΔR/R_0_ decreased when the temperature increased, exhibiting negative temperature coefficient (NTC) behavior [43]. The NTC effect is caused by the fact that an increase in temperature promotes the migration of charge carriers, resulting in a decrease in resistance. The temperature coefficient of resistance (TCR) was 0.321% according to the slope of the fitting curve, which showed outstanding temperature sensitivity [44]. ΔR/R_0_ is monotonic and linear in the temperature range of 25–50 °C. It was also found that the experimental data were in agreement with the theoretical curve (R^2^ = 0.999).

Furthermore, the temperature change of the sponge was detected by the resistance change of the sensor. Figure 6c shows a schematic diagram of detecting the resistance change of a sample irradiated by simulated sunlight. When irradiated by the xenon lamp, the sensor temperature quickly rose to 71 °C within 5 s. The carbon nanoparticle network was capable of converting light energy into heat energy. Some of the heat was used to raise the temperature. As a result, the resistance of the sensor was reduced. When the xenon lamp irradiation was removed, the temperature of the sensor gradually decreased, and the resistance returned to the initial value (Figure 6d). The temperature sensor responded quickly, with a response time of only 132.7 ± 2.1 ms. In addition, the intensity of sunlight also affected the change in sensor resistance. As shown in Figure 6e, the resistance changes of the sensor increased as the irradiation intensity increased, and the resistance change was stable (Figure 6f). Therefore, CS-ink can be utilized as a wearable temperature sensor to track temperature changes in the environment.

## 3. Materials and Methods

### 3.1. Materials

Zhujiang brand Chinese ink was purchased from Guangzhou Jinjian office manufacturing plant, China. Chitosan (deacetylation degree ≥ 95%, viscosity-average molecular weight of 600,000 g/mol, viscosity (10 g/L, 20 °C) of 200 mPa·s) was purchased from Jinan Haidebei Marine Bioengineering Co., Ltd., Jinan, China. Glacial acetic acid was obtained from Guangdong Guangshi Reagent Technology Co., Ltd., Shenzhen, China.

### 3.2. Preparation of Chitosan-Ink Carbon Nanoparticles Sponge

A 2 wt% chitosan solution was prepared in a 2 wt% glacial acetic acid solution and stirred for 12 h. Chinese ink (10%, 25%, and 50% relative to the mass of the chitosan solution) was added to the chitosan solution. The chitosan-ink solution was obtained after stirring for 1 h and being subjected to ultrasonic shock treatment. Then, the solution was poured into a mold and frozen at −70 °C. After freeze-drying for 48 h in a vacuum freeze-dryer (Ningbo Scientz Biotechnology Co., Ltd., Ningbo, China), chitosan-ink carbon nanoparticle sponges with a length, width, and height of 20 × 20 × 4 mm and a diameter of 15.6 mm and a height of 20 mm were obtained. Chitosan sponges with 25% ink content were named as CS-ink, and this sample was used for the following measurement. The preparation method of the pure chitosan sponge (named CS) was the same as above. Finally, all samples were placed in an oven at 60 °C for a period of time to remove the acid from the sponge.

### 3.3. Characterization Methods

The cross-section morphology of the sponges was examined using a scanning electrospinning microscopy (SEM, Zeiss Sigma 500, Oberkochen, Germany). Before observation, the surface of the samples was coated with a thin gold layer. The microstructure of carbon nanoparticles and CS-ink was observed with a transmission electron microscope (TEM, JEOL/JEM-1400 Flash, Tokyo, Japan). The chemical structure of chitosan and CS-ink was characterized using a Fourier transform infrared spectrometer (Nicolet iS50, Thermo Fisher Scientific Ltd., Waltham, MA, USA) in a scope of wavenumber from 4000 to 400 cm^−1^. The XRD patterns of CS and CS-ink were determined by an X-ray diffractometer (MiniFlex-600, Rigaku Corporation, Tokyo, Japan) at an accelerating voltage of 40 kV and a current of 40 mA. The diffraction angles were 5 to 60° with a 15°/min scan rate. From the measurements of the electrophoretic mobility, the zeta potentials of ink, CS, and CS-ink dispersions were calculated from the Henry equation using zetameter software via a Nano ZS zeta potential analyzer (Malvern Instruments Co., Malvern, UK). The thermolysis curves of CS and CS-ink sponges were analyzed by a thermogravimetric analyzer (Mettler Toledo, Co., Ltd., Zürich, Switzerland). About 10 mg of the sponge was placed in an Al_2_O_3_ crucible and heated from 30 to 600 °C at a heating rate of 10 °C/min under a nitrogen atmosphere. The first derivative of TG (DTG) was also analyzed by the software. The experimental data were processed and expressed by the mean values ± standard deviation (SD).

### 3.4. Mechanical Properties

A universal testing machine (AGS-X, Shimadzu, Japan) was conducted to measure the compression resistance of CS and CS-ink sponges at 25 °C and 55% RH. The samples (diameter of 15.6 mm, height of 7 mm) were compressed to 80% of their original volume by a longitudinal pressure at a 5 mm/min compression rate.

### 3.5. Porosity

The porosity of the sponges was analyzed by the absolute alcohol displacement method. The weight and size of the samples were measured first. Then, the dried samples were soaked in alcohol. After 6 h, the samples were taken out and weighed. Each group should be tested three times. The porosity was calculated using the following formula (Equation (1)),

(1) $$Porosity\;\left(\%\right)=\frac{m_{2}-m_{1}}{\rho \times V}\times 100\%$$

where *m*_2_ is the weight of the samples after immersing in alcohol, *ρ* is the density of alcohol (0.79 g/cm^3^ at room temperature), and *m*_1_ and *V* are the mass and volume of the samples at the initial state, respectively.

### 3.6. Electrical Conductivity

The conductivity of the cylindrical samples was measured using a multimeter (Fluke 17B+, Fluke Electronic Instrument Ltd., Everett, WA, USA). The sample’s left and right ends were wrapped with copper wires, and the other copper wires were connected to a multimeter. The conductivity was calculated as follows:

(2) $$G\;\left(S/m\right)=\frac{L}{R\times S}$$

where *G* is the conductivity, *L* is the length of the sample in the current direction, *R* is the resistance, and *S* is the cross-sectional area of the sample perpendicular to the current direction.

### 3.7. Photothermal Performance

The samples (diameter of 15.6 mm and height of 20 mm) were irradiated by an 808 nm fiber-coupled laser (MD-III-808, Changchun New Industry Optoelectronic Technology Ltd., Changchun, China). The temperature changes of the chitosan sponges with different ink contents were measured by irradiating them at 0.7 W/cm^2^ power density for 5 min. The surface temperatures and images of the CS-ink sponge were recorded by an infrared imager (TIS 55, Fluke Electronic Instrument Ltd., Everett, WA, USA) in real time. The power density of the laser was adjusted in the range of 0.5–0.9 W/cm^2^, and the measuring distance between the laser and the sample was 1 cm. The parallel test was done three times.

### 3.8. The Application of CS-Ink Sponge as a Strain Sensor

The sponge with a size of 20 × 20 × 4 mm was attached to the fingers, wrists, elbow, spine, and throat of the human body with polyimide tape. One end of the copper wire was connected to the sponge and the other to the electrometer (Keithley 6514, Tektronix Inc., Beaverton, OR, USA). Subsequently, the signal response was recorded using an electrometer by pressing the sample; bending the finger, wrist, and elbow; nodding; and swallowing. Three groups of parallel samples were tested for each action, and each test cycle was repeated several times.

### 3.9. Temperature Monitoring

The CS-ink sponge adhered to the outer wall of a beaker filled with 450 mL of silicone oil. The sponge was connected to the multimeter by copper wire. The temperature of the sensor was changed by heating the silicone oil, and the resistance change was measured by a multimeter.

The temperature of the sensor was varied by changing the power of simulated sunlight (Oriel Class A solar simulator, Oriel 91195A, Newport Co., Irvine, CA, USA). The sponge under simulated sunlight was connected to the electrometer through wires. The wires were taped to the table to reduce errors caused by vibration. The xenon lamp was turned on to irradiate the sample for 5 s and then turned off for 5 s, cycling the process several times. The relationship between the signal response from the sensor and temperature was measured. The measurement distance between the xenon lamp and the sample was 16 cm. The parallel test was done three times.

## 4. Conclusions

In conclusion, a flexible temperature–pressure response sensor with excellent photothermal properties and good electrical conductivity was developed. The sensor consists of Chinese ink and chitosan, obtained by a simple solution blending and freeze-drying method. The carbon nanoparticles in ink impart good conductivity and superior photothermal conversion properties to the chitosan sponge, with a conductivity of 0.1565 S/m and a rapid temperature rise to 254.7 °C under NIR irradiation at low power density (0.9 W/cm^2^) within 5 s. Additionally, the composite sponge was used as a strain–temperature sensor for the detection of human motion and temperature changes in real time. The average response time of the sensor is 133.05 ms, which provides a fast output of stable and repeatable signals. Therefore, CS-ink sensors have a wide range of application prospects in wearable electronics, electronic skin, health monitoring, environmental temperature detection, and other fields.

## Data Availability

All the data have been included in the study.
